# Ferumoxytol-enhanced MRI in patients with prior cardiac transplantation

**DOI:** 10.1136/openhrt-2019-001115

**Published:** 2019-10-03

**Authors:** Colin G Stirrat, Shirjel Alam, Thomas J MacGillivray, Calum Gray, Marc Richard Dweck, Victor Jones, William Wallace, John R Payne, Sanjay K Prasad, Roy S Gardner, Mark C Petrie, Saeed Mirsadraee, Peter Henriksen, David E Newby, Scott Semple

**Affiliations:** 1 Centre for Cardiovascular Science, University of Edinburgh, Edinburgh, UK; 2 Department of Cardiovascular Sciences, University of Edinburgh, Edinburgh, UK; 3 Clinical Research Imaging Centre, University of Edinburgh, Edinburgh, UK; 4 Department of Pathology, University of Edinburgh, Edinburgh, UK; 5 Scottish National Advanced Heart Failure Service (SNAHFS), Golden Jubilee National Hospital, Clydebank, UK; 6 Department of Cardiology, Royal Brompton Hospital, London, UK; 7 Scottish Advanced Heart Failure Unit, Golden Jubilee National Hospital, Clydebank, UK; 8 Institute of Cardiovascular and Medical Sciences, University of Glasgow, Glasgow, UK; 9 Golden Jubilee National Hospital, Clydebank, UK; 10 Edinburgh Heart Centre, Royal Infirmary of Edinburgh, Edinburgh, UK

**Keywords:** cardiac, MRI, cardiac transplant, inflammation, USPIO

## Abstract

**Objectives:**

Ultra-small superparamagnetic particles of iron oxide (USPIO)-enhanced MRI can detect cellular inflammation within tissues and may help non-invasively identify cardiac transplant rejection. Here, we aimed to determine the normal reference values for USPIO-enhanced MRI in patients with a prior cardiac transplant and examine whether USPIO-enhanced MRI could detect myocardial inflammation in patients with transplant rejection.

**Methods:**

Ten volunteers and 11 patients with cardiac transplant underwent T2, T2* and late gadolinium enhancement 1.5T MRI, with further T2* imaging at 24 hours after USPIO (ferumoxytol, 4 mg/kg) infusion, at baseline and 3 months.

**Results:**

Ten patients with clinically stable cardiac transplantation were retained for analysis. Myocardial T2 values were higher in patients with cardiac transplant versus healthy volunteers (53.8±5.2 vs 48.6±1.9 ms, respectively; p=0.003). There were no differences in the magnitude of USPIO-induced change in R2* in patients with transplantation (change in R2*, 26.6±7.3 vs 22.0±10.4 s^-1^ in healthy volunteers; p=0.28). After 3 months, patients with transplantation (n=5) had unaltered T2 values (52.7±2.8 vs 52.12±3.4 ms; p=0.80) and changes in R2* following USPIO (29.42±8.14 vs 25.8±7.8 s^-1^; p=0.43).

**Conclusion:**

Stable patients with cardiac transplantation have increased myocardial T2 values, consistent with resting myocardial oedema or fibrosis. In contrast, USPIO-enhanced MRI is normal and stable over time suggesting the absence of chronic macrophage-driven cellular inflammation. It remains to be determined whether USPIO-enhanced MRI may be able to identify acute cardiac transplant rejection.

**Trial registration number:**

NCT02319278349 (https://clinicaltrials.gov/ct2/show/NCT02319278) Registered 03.12.2014 EUDraCT 2013-002336-24.

**Key questions**

**What is already known about this subject?**

- Ultra-small superparamagnetic particles of iron oxide (USPIO) are ingested by tissue macrophages that can be visualised using MRI to highlight areas of macrophage inflammation within the heart.

**What does this study add?**

- Stable patients with cardiac transplantation have increased myocardial T2 values, consistent with resting myocardial oedema or fibrosis. Despite this, USPIO-enhanced MRI is normal and stable over time suggesting the absence of chronic macrophage-driven cellular inflammation.

**How might this impact on clinical practice?**

- USPIO-enhanced T2* MRI may still prove to be of value in diagnosing and monitoring conditions with macrophage-driven myocardial inflammation, including acute transplant rejection with associated macrophage infiltration.

## Introduction

Cardiac transplantation is a life-prolonging treatment for end-stage cardiac disease. Transplant rejection is a major threat to the allograft, requiring treatment in around one in eight transplant recipients in the first year^1^ but can occur at any stage after transplantation and causes significant morbidity and mortality. Rejection is notoriously difficult to diagnose using existing non-invasive imaging methods with repeated surveillance myocardial biopsies often undertaken.

Most cases of acute rejection are due to cellular rejection with antibody-mediated rejection less prevalent. Rejection severity is classified according to histological findings, and although the cellular infiltrate in acute cellular rejection is predominantly lymphocytic, macrophage infiltration has a key role.^2 3^ The importance of macrophages in acute cardiac allograft rejection was recently emphasised in a rodent study that showed depletion of circulating macrophages protected the allograft against rejection, raising the possibility of therapeutic targeting of macrophages as a novel treatment strategy.^4^

Iron oxide nanoparticles are generating interest as a MRI contrast medium that is able to detect macrophages, and clinical applications, such as myocardial infarction, are now emerging.^5–11^ Ultra-small superparamagnetic particles of iron oxide (USPIO) consist of an iron oxide core surrounded by a carbohydrate or polymer coating. They are small enough to extravasate passively through capillaries, where they are engulfed by tissue-resident macrophages^12^ and are detectable by T2*-weighted MRI. Thus, USPIO-enhanced MRI can identify tissue-resident macrophage activity and help to identify cellular inflammation within tissues.

Promising preclinical studies have shown USPIO-enhanced MRI is able to detect acute cardiac and renal allograft rejection with USPIO signal correlating with macrophage distribution, rejection severity on histology and impaired cardiac function. Moreover, this approach can also be used to assess treatment response with rodent models demonstrating less USPIO enhancement following initiation of immunosuppression.^4 13–17^ A future role of USPIOs includes a ‘theranostic’ strategy whereby imaging is combined with therapy; Guo *et al*^18^ recently conjugated an iron nanoparticle to a CD-3 antibody and a therapy gene, allowing imaging and targeting of T cells that play a central role in acute cardiac allograft rejection.

In this study, we aimed to assess and quantify myocardial USPIO enhancement in stable patients with cardiac transplantation and patients with cardiac transplant rejection, correlating enhancement with clinical measures of inflammation and oedema including T2 mapping MRI,- a quantitative imaging method assessing myocardial oedema in transplant rejection.^19 20^ We hypothesised that USPIO-enhanced MRI would detect myocardial macrophage activity in the inflamed myocardium of rejecting transplanted hearts, but not in stable healthy cardiac allografts, and provide a cellular-specific non-invasive imaging technique that may aid and improve patient diagnosis and management.

## Methods

This was an open-label observational multicentre cohort study (NCT02319278) that recruited patients between January 2015 and May 2016. The study was performed in accordance with the Declaration of Helsinki, the approval of the Scotland A Research Ethics Committee (13-SS-0111), and the written informed consent of all participants. The Medicines and Healthcare products Regulatory Authority of the United Kingdom gave Clinical Trial Authorisation for the study (EUDraCT 2013-002336-24).

### Study populations

Adult (>18 years of age) patients with a history of cardiac transplantation (including suspected allograft rejection) were recruited into the study. Healthy volunteers had no clinically significant medical history. Exclusion criteria were contraindication to MRI or ferumoxytol infusion, any other inflammatory comorbidity, renal failure (estimated glomerular filtration rate <30 mL/min/1.73 m^2^), pregnancy, breastfeeding and women of childbearing potential without reliable contraception.

### Study protocol

Patients with cardiac transplantation and healthy volunteers underwent paired MRI scans at baseline, and patients were invited to return for repeat imaging after 3 months. At the time of scanning, blood samples were collected for clinical haematology and biochemistry measurements.

### Magnetic resonance imaging

MRI was performed using a MAGNETOM Avanto 1.5T MRI (Siemens Healthcare, Erlangen, Germany), with a dedicated cardiac array coil. All images were acquired with ECG gating using expiration breath-holds. Routine steady state free precession (TrueFISP) sequences were used to acquire long-axis and short-axis cine images of the heart (repetition time (TR) 85.8 ms, echo time (TE) 1.45 ms, flip angle 50°, matrix 173×256, field of view (FoV) 400 mm, slice thickness 8 mm, 2 mm gap). Quantitative USPIO imaging was performed in similar slice positions using a prototype T2*-weighted multigradient-echo acquisition with a volumetric shim applied over the entire heart volume (TR 996 ms, TE 2.13, 4.3, 6.4, 8.6, 10.7, 12.8, 15.0, 17.1 ms, flip angle 18°, matrix 130×256, FoV 400 mm, slice thickness 6 mm, gap 4 mm). The T2*-weighted acquisitions included views through the liver, spleen and spine to allow quantification of USPIO accumulation within organs of the reticuloendothelial system. The same T2* protocol was used to quantify USPIO accumulation 24 hours after infusion allowing calculation of T2* relaxation rates before and after administration of USPIO. T2 mapping was conducted using a Siemens prototype T2-prepared TrueFISP acquisition acquiring identical long-axis and short-axis slice positions (TR 219.3 ms, TE 1.07 ms, T2 prep durations 0, 25, 50 ms, flip angle 70°, matrix 130×192, FoV 400 mm, slice thickness 6 mm, 4 mm gap). T2P-TrueFisp images are acquired at intervals of at least three RR intervals to allow for sufficient magnetisation recovery in between acquisitions.

Immediately after the baseline T2 and T2*-weighted scan, participants received an intravenous administration of gadolinium contrast medium (0.15 mmol/kg; Gadovist, Bayer Plc, Germany) followed by breath-held inversion recovery sequences in long-axis and short-axis planes to acquire late-enhancement images. Optimal inversion time (TI) was determined on a slice-by-slice basis using standard late-enhancement TI-scout protocols (TR 750 ms, TE 2.61 ms, flip angle 20°, matrix 173×256, FoV 400 mm, slice thickness 9 mm, gap 1 mm). The inversion-recovery late-enhancement short-axis slices were acquired using similar slice positions as the T2-oedema and T2*-weighted imaging.

### Ultra-small superparamagnetic particles of iron oxide

Intravenous infusion of USPIO (ferumoxytol, 4 mg/kg; Rienso, Takeda Italia, Italy) was performed immediately following the baseline magnetic resonance scan over 15 min using a concentration of 2–8 mg/mL, diluted in 0.9% saline or 5% dextrose. Haemodynamic monitoring was conducted throughout, and participants were observed for a further 30 min to ensure no hypersensitivity reactions.

### Image analysis

All T2*-weighted multigradient-echo images for each patient were analysed using Circle CVI software (Circle CVI42, Canada). An experimentally determined threshold used in previous work^6^ for the coefficient of determination (r^2^ >0.85) was used to exclude data that did not have an acceptable exponential decay when signal intensity (SI) was plotted against echo time. Individual images affected by artefact were excluded. The inverse of the mean T2* (R2*) for each region of interest (ROI) was then calculated to assess the uptake of USPIO, where the higher the value, the greater the USPIO accumulation.

**Figure 1:**
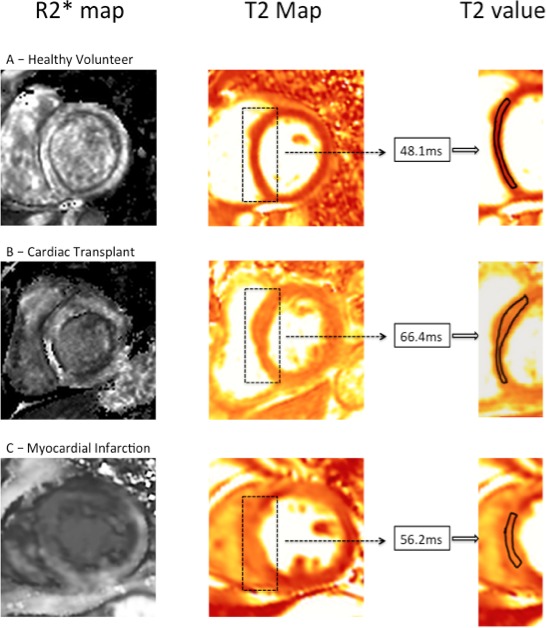
Post-USPIO R2* and T2 maps for a healthy volunteer (A), patient with cardiac transplant (B) and myocardial infarction (C). Myocardial oedema is displayed as brighter signal in the T2 colour map corresponding to higher T2 value. Cardiac transplant myocardium (B) displays no USPIO accumulation but high T2, in contrast to USPIO accumulation and high T2 in myocardial infarction (C). USPIO, ultra-small superparamagnetic particles of iron oxide.

T2 maps, ventricular volume and functional analyses were also performed using Circle CVI software (Circle CVI42, Canada). T2 and T2* data were collected immediately prior to USPIO administration. USPIO-enhanced T2* data were collected 24–25 hours following ferumoxytol administration. ROIs were drawn on T2 and T2* images in the septum at midcavity level between anterior and inferior RV insertion points (see figure 1). Septal myocardial regions only were selected to reduce influence of artefact caused by nearby stomach or lung.^21^

### Histology

Myocardial tissue samples were obtained from one patient with cardiac transplantation undergoing surveillance biopsies without suspicion of transplant rejection. USPIO was administered 24 hours prior to the biopsy, and a trucut myocardial biopsy sample was taken from the myocardium. The biopsy sample was fixed in formalin, embedded in paraffin, sectioned and stained to look at architecture (H&E), accumulation and distribution of USPIO (Prussian Blue) and macrophages (CD68).

### Sample size and statistical analysis

Previous work has shown the change in R2* due to USPIO in ‘healthy’ myocardium (remote from the site of myocardial infarction) to be 40±11 s^-1^ at 24 hours following USPIO.^6^ Assuming the change due to USPIO is similar in truly healthy myocardium, a sample size of 10 in each group is needed to detect an effect size of 20 s^-1^ due to USPIO (ie, an increase of 50%) with 80% power and significance level of 0.05.

All statistical analysis was performed with GraphPad Prism, version 6 (GraphPad Software, San Diego, California, USA). Shapiro-Wilk or D’Agostino and Pearson tests were used to test normality of distribution. To compare participant characteristics, USPIO uptake and myocardial oedema in patients and volunteers, R2* and T2 values were compared using χ^2^, unpaired t-tests and Mann-Whitney tests as appropriate depending on normality distribution. To compare results at 3 months with baseline, paired t-test and Wilcoxon test was used. Statistical significance was defined as two sided p<0.05.

## Results

Ten volunteers and 11 patients with cardiac transplantation were recruited. One volunteer was excluded from analysis due to the presence of LGE as described previously.^21^ At the time of recruitment, all transplant patients were assessed to be clinically well with no firm evidence of allograft rejection based on clinical history, examination and routine blood samples. One patient with prior cardiac transplant was excluded due the finding of LGE consistent with myocardial infarction that may influence the USPIO enhancement data.^6^ No other patient with cardiac transplant displayed LGE. Five of the 10 cardiac transplant patients returned at 3 months for repeat imaging. Administration of ferumoxytol was well tolerated with no adverse reactions reported during or immediately after administration in any of the participants.

Volunteers were predominantly female and patients with cardiac transplant were predominantly male (table 1). Transplant patients had smaller indexed end-diastolic ventricular volumes (p<0.01) and higher baseline plasma high-sensitivity troponin concentrations (p<0.05). There were more males in the transplant group (p<0.05), but there were no other differences between groups at baseline.

**Table 1:** Participant characteristics

	Healthy volunteers	Patients with transplant
Number	9	10
Female	6	1^*^
Age (years)	52 (45.5–61.5)	60 (52.75–64.5)
Time since transplantation (months)		59 (19–159)
Body mass Index (kg/m^2^)	22.9 (20.1–26.9)	25.9 (24.0–27.9)
Left ventricular end-diastolic volume (mL/m^2^)	80.9±10.4	62.9±15.5**
Left ventricular ejection fraction (%)	63.6±4.9	65.0±7.1
Blood tests		
White cell count (×10^9^/L)	6.5±1.5	6.1±2.5
C reactive protein (mg/L)	1.9±2.0	2.1±2.1
Plasma troponin (ng/L)	3.1±2.8	14.1±20.9*

Mean±SD or median (IQR).

*p<0.05, **p<0.01 (compared with volunteers).

### T2 and R2* mapping

**Figure 2:**
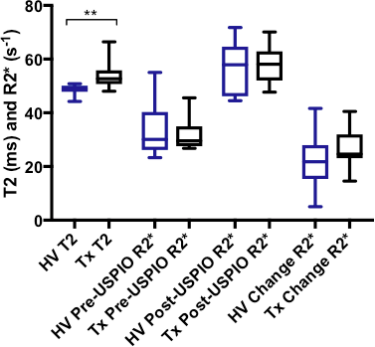
Comparison of T2 (ms) and R2*(s^-1^) measurements at baseline in healthy volunteers (HV) and cardiac transplant patients (Tx). Transplant patients have higher T2 than healthy volunteers, but no other significant differences exist between groups (**p<0.01). USPIO, ultra-small superparamagnetic particles of iron oxide.

Patients with cardiac transplantation had a higher T2 value than volunteers (53.8±5.2 vs 48.6±1.9 ms, respectively, p=0.003; figures 1 and 2 and table 2). There were no differences in R2* between patients with cardiac transplantation and volunteers at baseline (31.6±5.9 vs 34.0±10.1 s^-1^, p=0.84), at 24 hours after USPIO administration (58.2±7.5 vs 56.0±10.2 s^-1^, p=0.60) or in the magnitude of change in R2* due to USPIO (26.6±7.3 vs 22.0±10.4 s^-1^, p=0.28; figures 1 and 2 and table 2).

**Table 2:** Septal myocardial R2* and T2 values in healthy volunteers and patients with cardiac transplantation

	Patients with transplant	Healthy volunteers
Pan-myocardial pre-USPIO R2* (s^-1^)	31.6±5.9	34.0±10.1
Pan-myocardial post-USPIO R2* (s^-1^)	58.2±7.5	56.0±10.2
Pan-myocardial ΔR2* (s^-1^)	26.6±7.3	22.0±10.4
T2 (ms)	53.8±5.2*	48.6±1.9

Mean±SD.

*p<0.01 (compared with volunteers).

USPIO, ultra-small superparamagnetic particles of iron oxide.

**Figure 3:**
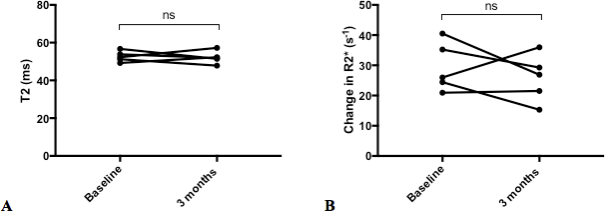
Repeated imaging in patients with cardiac transplant. There were no differences in myocardial T2 (ms) (A) or the change in R2* (s^-1^) (B) due to USPIO between time points (n=5).

**Figure 4:**
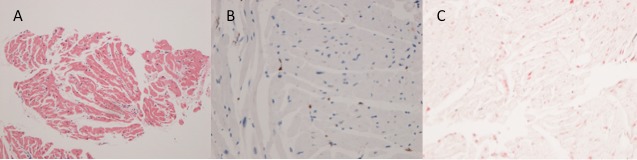
Biopsy from patient with cardiac transplant. (A) H&E (×100) staining showing normal myocyte architecture with no evidence of significant inflammatory cell infiltration. (B) CD68 staining (×200) showing only very few scattered macrophages (brown). (C) Prussian blue staining showing no evidence of iron.

There were no differences in either T2 (52.7±2.8 vs 52.12±3.4 ms, p=0.80) or the change in R2* due to USPIO (29.42±8.14 vs 25.8±7.8 s^-1^, p=0.43) between baseline and 3 months in patients with cardiac transplant (figure 3). The myocardial biopsy 24 hours after USPIO administration revealed normal myocyte architecture with no evidence of increased numbers of inflammatory macrophages or the presence of USPIO (figure 4).

### Cardiac transplant patient with recently suspected allograft rejection

One patient was recruited to the study 3 days following admission with non-specific malaise. This was the only patient in our cohort that had a previous history of previous acute cellular rejection (2 years before). On this occasion, the myocardial biopsy showed grade 1a rejection (ie, no evidence of recurrence of rejection or cellular infiltration), and the patient was reassured but subsequently recruited to the study. This patient had the highest plasma high-sensitivity troponin I in the cohort at 71 ng/L. Other blood tests were normal including white cell count (4.3×10^9^/ L) and C reactive protein (<1 mg/L). The T2 value for this subject was the highest in our cohort at 66.4 ms (16-segment average of 61.8 ms). R2* was 26.7 s^-1^ at baseline and 50.6 s^-1^ 24 hours after USPIO: change in R2* was 23.8 s^-1^. A reanalysis of the data censoring this patient from analysis continued to show a higher T2 value in patients with cardiac transplantation than volunteers (52.4±2.8 vs 48.6±1.9 ms, p=0.0056). This patient did not undergo gadolinium-enhanced imaging at the request of the attending clinician due to mild renal impairment.

## Discussion

For the first time, we report the combined assessment of T2 mapping and USPIO-enhanced T2* MRI in patients with prior cardiac transplantation and healthy volunteers. This technique is feasible, safe and well tolerated in this group of patients. We demonstrate that while myocardial T2 is increased, there is no evidence of ongoing or chronic cellular inflammatory uptake as detected by USPIO enhancement. Further assessments of these measures are now needed in patients with acute cardiac transplant rejection.

USPIO-enhanced T2* MRI has previously been used in man to assess cardiovascular inflammation in a range of different conditions,^5–8 11^ and preclinical models suggest this technique may be useful in assessing human patients with transplant rejection.^4 13–17^ In our cohort of stable patients with cardiac transplantation, we did not detect greater USPIO enhancement within the myocardium, but this contrasted with the increased measures of myocardial T2 found in transplant patients compared with volunteers.

Why did we detect higher T2 values in our cohort of transplant patients compared with controls in the absence of USPIO accumulation? There are several plausible explanations. First, chronic residual low-grade inflammation or oedema without tissue-resident macrophage infiltration is possible, perhaps from lymphocytes that do not take up USPIO. In the absence of histological data, we cannot confirm the presence of lymphocytes, but in a stable group of patients who are clinically well, this explanation appears unlikely. Second, higher T2 values in the myocardium of cardiac allografts may simply be due to increased water content, possibly related to expansion of the myocardial vasculature and blood content. The finding of greater measures of R2* (both at baseline and after USPIO), which measures iron within the blood pool, would support this mechanism. This explanation may suggest intriguing differences in the behaviour of resting myocardial vascular integrity in patients with stable cardiac allografts. Third, the relatively low field strength of the MRI scanner (1.5T) may be contributory since it has poorer sensitivity in detecting sparse myocardial USPIO compared with higher field strength 3T scanners. However, in our single myocardial biopsy, substantial numbers of macrophages were not evident. Finally, a possible explanation for greater measures of T2 in cardiac allografts may in fact reflect a change in myocyte architecture caused by myocardial fibrosis or scarring. High T2 value is usually solely attributed to myocardial oedema but has previously been found to inversely correlate with LV function in patients with dilated cardiomyopathy that did not have any evidence of inflammation or other reasons for myocardial oedema.^22^ This may suggest that in this cohort higher T2 values reflect myocardial fibrosis, similar to results using T1 mapping techniques. Recently published data in a similar cohort of patients found elevation of T2 and T1 values in all transplanted patients, even without history of cardiac rejection, and the authors suggest this is due to greater myocardial oedema and interstitial fibrosis at baseline.^23^ However, we contend that elevated T2 in the myocardium of cardiac allografts in this cohort may reflect intrinsic changes to the cellular architecture, such as the presence of myocardial fibrosis and not myocardial oedema. As such, it may prove to be a further imaging biomarker of myocardial fibrosis. Crucially, we lacked adequate access to sufficient histology or T1 mapping in order to prove whether subtle myocardial fibrosis, not detected by LGE imaging, is a causative factor in the high T2 value found. Most patients had undergone transplantation many years ago (see table 1) so using historical postoperative surveillance biopsies would not provide an accurate assessment of current myocyte architecture.

Irrespective of the aetiology, greater T2 in stable transplant patients is an interesting finding reported recently that we have now confirmed and warrants further investigation. Previous small studies have found that T2-weighted and T2 mapping MRI assists in diagnosis and prognosis estimation in patients with cardiac transplant rejection.^24–26^ Following on from these studies, the DRAGET (Detection of Acute Graft Rejection in Heart Transplant Patients by Estimation of T2) study,^27^ a large multicentre multinational study, is now prospectively recruiting patients in the first year after transplantation to assess the performance of T2 mapping in diagnosing transplant rejection. The findings of this study are awaited with interest, but clearly if the T2 value in myocardium of stable cardiac allografts is raised, this increases the likelihood of false-positive diagnoses of allograft rejection and ultimately reduces the specificity and precision of the test.

One patient had very high myocardial T2 and deserves special mention. This was the only patient with a previous history of biopsy proven acute cellular rejection 2 years previously. At the time of recruitment, the patient was recovering from a non-specific illness, and recurrent episode of rejection had been discounted on the basis of negative endomyocardial biopsies 3 days previously. Based on the studies mentioned previously, myocardial T2 value of this magnitude suggests severe rejection. Serial imaging with T2 mapping, both prior and subsequent to this episode, would have been useful in determining the time course and fluctuation in T2 to assess whether it is chronically elevated, which may suggest stable myocardial fibrosis or not. This patient in fact had T1 maps acquired as part of imaging biobank retention (the only one in our study), and septal values were elevated at 1018 ms (normal values 950±21 ms).^28^ Clearly, we cannot make generalisations from one patient, but this provides further support to the argument that high T2 may reflect myocardial fibrosis.

Whether this patient had allograft rejection (with a negative biopsy) remains uncertain, but a clinical improvement was made with no new changes to medical therapy, making an episode of rejection is less likely. There was certainly no evidence for USPIO uptake or significant macrophage infiltration. If myocardial fibrosis and active rejection or inflammation is not the cause of elevated T2, then why was the T2 value so high in this patient? A high T2 value of this magnitude in the absence of rejection would cast doubt on the precision of T2 mapping to diagnose and exclude cardiac allograft rejection and diminish its potential utility in clinical practice. The DRAGET study will be well placed to answer this.^27^

There are some limitations that deserve mention. Our numbers were small but sufficient for a pilot study testing the feasibility of USPIO-enhanced MRI in this population. From our recent work in patients with myocardial infarction, we know that this technique works and is capable of identifying and tracking myocardial macrophage accumulation macrophage.^5^ In this study, myocardial macrophage infiltration is either not a chronic feature of stable transplants or at levels too low to be detected.

We intended to recruit a range of patients with cardiac transplantation, from chronic stable patients, to patients with suspected allograft rejection. Unfortunately, we were only able to recruit one patient with recently suspected transplant rejection, and this was excluded clinically, with the remaining patients being stable and well. Small study numbers and absence of patients with florid transplant rejection clearly limit our findings, and this technique now needs to be tested in patients with proven acute transplant rejection.

In conclusion, we have shown that stable patients with cardiac transplantation have greater measures of myocardial T2, which may suggest myocardial oedema or fibrosis, compared with control volunteers. Ferumoxytol-enhanced MRI does not add clinically relevant information in this group of stable patients, and it remains to be determined whether this technique may be able to identify macrophages in the setting of proven acute cellular transplant rejection.
